# Radiological and Clinical Outcome Differences Between Standard and Short Stem in Reverse Total Shoulder Arthroplasty: A Systematic Review

**DOI:** 10.3390/medsci13010016

**Published:** 2025-02-09

**Authors:** Mauro Ciuffreda, Antongiulio Lentini, Giuseppe Francesco Papalia, Domenico Grasso, Pierangelo Za, Rocco Papalia, Giacomo Rizzello

**Affiliations:** 1 Department of Orthopedic and Trauma Surgery, Università Campus Bio-Medico di Roma, Via Alvaro del Portillo, 21, 00128 Roma, Italy; m.ciuffreda@policlinicocampus.it (M.C.); antongiulio.lentini@unicampus.it (A.L.); domenico.grasso@unicampus.it (D.G.); za.pierangelo@gmail.com (P.Z.); r.papalia@policlinicocampus.it (R.P.); g.rizzello@policlinicocampus.it (G.R.); 2 Research Unit of Orthopedic and Trauma Surgery, Fondazione Policlinico Universitario Campus Bio-Medico, Via Alvaro del Portillo, 200, 00128 Roma, Italy

**Keywords:** reverse shoulder arthroplasty, cementless, short stem, standard stem, radiological outcomes, clinical outcomes

## Abstract

Background: In recent years, the use of short cementless humeral components in reverse total shoulder arthroplasty (RTSA) has increased. This systematic review aimed to compare the radiological and clinical outcomes of uncemented RTSA using short versus standard humeral stems and assess the impact of these radiological changes on clinical outcomes. Methods: A systematic electronic search was performed by two independent reviewers using PubMed, Scopus, and Cochrane Library databases on 10 December 2024. Inclusion criteria involved studies that assessed the radiological and clinical outcomes and overall complication rates of cementless RTSA with short or standard stems in patients with osteoarthritis, cuff tear arthropathy, post-traumatic, and rheumatoid arthritis with a follow-up of at least 1 year. The following data were extracted: radiological parameters of stems including implant subsidence, humeral loosening, and humeral osteolysis and clinical outcomes as Visual Analog Scale pain, American Shoulder and Elbow Surgeons score, Constant Score and Single Assessment Numeric Evaluation score. Results: A total of 13 studies including 1485 shoulders in 1460 patients were analyzed with a median age at surgery of 74.5 years. The short stem group recorded worse radiological outcomes examined such as humeral loosening, lucencies around the implants, and osteolysis. No significant differences were observed in the clinical outcomes and overall complications between the two types of stems. Conclusions: Both short and standard stems are valid options in cementless RTSA. Minimal differences in radiological outcomes were found in favor of RTSA implanted with short stems, while postoperative clinical outcomes were similar between the two types of implants.

## 1. Introduction

Reverse total shoulder arthroplasty (RTSA) is a well-established treatment for end-stage arthritis, irreparable rotator cuff lesions, fractures or their sequelae in the proximal humerus, inflammatory arthritis, anatomical arthroplasty failure, or tumor-related conditions [1,2]. The surgical technique for RTSA requires securing the humeral component in the proximal humerus, with or without cement fixation, ensuring the capacity for osseous ingrowth. Although both methods are effective, their long-term impact on implant loosening remains uncertain [3]. RTSA has shown clinical success, with an overall implant survival rate of 94.5% at 2 years and a 4% overall complication rate at 90 days [4]. Complications have significantly reduced with advancements in implant design and scapular notching reduction over the years. Despite comparable clinical and radiographic outcomes between cemented and cementless prostheses, uncemented stems offer advantages such as reduced operative time, no risk of cement-related complications, and ease of revision [5,6]. The rise in cementless RTSA has led to increased clinical and radiological complications over time including radiolucent lines, osteolysis, and humeral stress shielding [7,8].

In recent years, the use of short cementless humeral components in shoulder arthroplasty has increased [4,9]. Short stems offer benefits such as bone stock preservation, ease of revision, safe placement in congenital or post-traumatic deformities, and reduced risk of diaphyseal stress risers, but concerns exist including potential malpositioning and higher mechanical failure rates [10,11] due to increased reliance on metaphyseal support alone [12].

Radiographic outcomes of cementless RTSA with short and standard stems remain unclear and are influenced by factors like mechanical stress shielding and biological reactions to debris from polyethylene insert and metal component degradation [13].

This systematic review aims to compare the radiological and clinical outcomes of uncemented stems in RTSA using short versus standard humeral stems with a follow-up of at least 1 year. We hypothesize that short humeral stems will demonstrate comparable outcomes to standard humeral stems in RTSA.

## 2. Materials and Methods

In adherence to the Preferred Reporting Items for Systematic Reviews and Meta-Analyses (PRISMA) guidelines, a comprehensive and systematic literature review was conducted on 10 December 2024 by two independent reviewers (A.L. and D.G.). The following keywords were used on PubMed, Cochrane Library, and Scopus databases: reverse total shoulder arthroplasty AND stem AND outcomes.

The initial phase involved the screening of articles for relevance based on title and abstract, with subsequent retrieval of full-text articles for further evaluation. In instances of disagreement, the senior investigator (M.C.) made the final decision.

Inclusion criteria were randomized controlled trials (RCTs), prospective, and retrospective comparative studies, and case series, written in English, that assessed the radiological and clinical outcomes of cementless RTSA with short or standard stems in patients with osteoarthritis, cuff tear arthropathy, post-traumatic and rheumatoid arthritis, and condrolysis with a follow-up of at least 1 year.

Conversely, exclusion criteria included studies involving infections, incomplete follow-up, revision arthroplasty, post-instability arthritis, proximal humerus fractures, cemented RTSA, and glenoid radiological outcomes. Moreover, articles failing to report the diagnosis, follow-up, or statistical analysis of radiological outcomes were also excluded.

From the selected articles, two independent authors (A.L. and G.F.P.) extracted the radiological parameters of stems including implant subsidence, humeral loosening, and humeral osteolysis as well as clinical outcomes as Visual Analog Scale (VAS) pain, American Shoulder and Elbow Surgeons (ASES) score, Constant Score (CS), Single Assessment Numeric Evaluation (SANE) score, and overall complication rates.

The primary endpoint focused on comparing the rate of subsidence, osteolysis, and humeral radiolucencies, stratified by different types of implants and stems (standard or short stem). The secondary endpoint involved comparing the rate of clinical outcomes and overall complications between the two groups of stems.

## 3. Results

**Figure 1:**
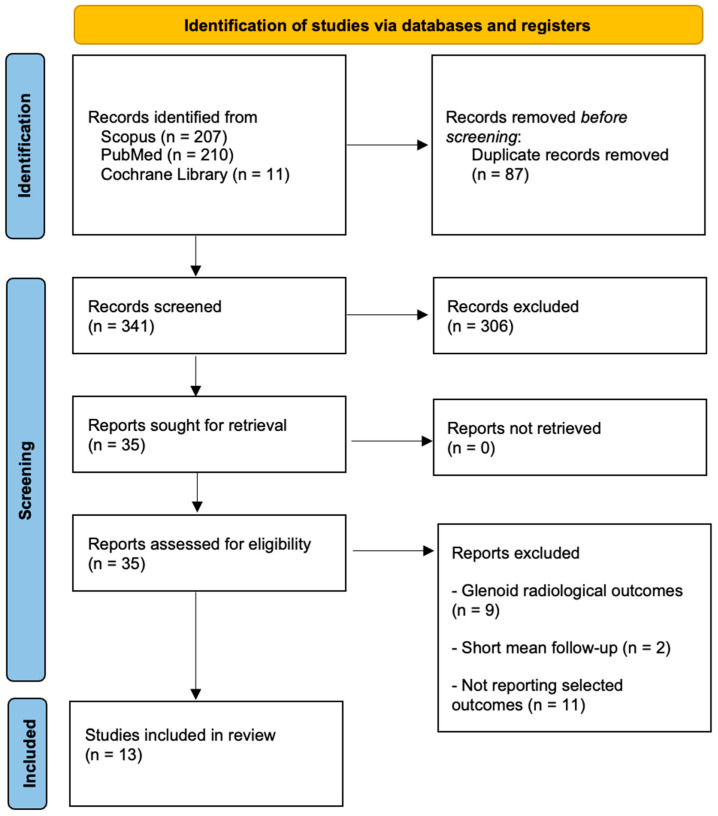
PRISMA 2020 flow diagram.

The search across databases yielded a total of 428 references. After removing 87 duplicates and excluding 306 articles based on predefined criteria, 35 articles were assessed for eligibility. Of these, nine were excluded due to glenoid radiological outcomes, eleven for not reporting selected outcomes, and two for short follow-up. The final selection included thirteen articles, comprising nine retrospective cohort studies, three case series, and one case–control study. The detailed process of study selection is presented in Figure 1.

### 3.1. Demographics

A total of 1485 shoulders in 1460 patients were included, with a median age at surgery of 74.5 years, ranging from 40 to 85 years (Table 1). Primary shoulder osteoarthritis was the main indication for arthroplasty, with other indications including cuff tear arthropathy, chondrolysis after arthroscopy, rheumatoid arthritis, and post-traumatic arthritis. A total of 989 shoulders received an uncemented standard implant by either DePuy Orthopaedics (Delta III; Warsaw, IN, USA) or Tornier (Aequalis Reversed Shoulder; Edina, MN, USA), and 496 shoulders received an uncemented RTSA with a short stem (Apex or Ascend Flex Tornier). In seven studies, standard stems were evaluated, while three studies presented short stems, and two studies assessed both stem types. The studies involved 1026 standard stems and 459 short stems. In the short stems group, 28 36-mm and 9 42-mm glenospheres were implanted. In the standard stems group, 48 36-mm glenospheres and 16 40-mm glenospheres were implanted. Patients were assessed at a follow-up period from 12 to 58 months for the standard group and from 12 to 240 months for the standard group.

### 3.2. Radiological Outcomes

All studies examined the radiological outcomes through X-ray studies during the final follow-up, predominantly at 48–60 months post-surgery (Table 2). Significant humeral loosening was identified in seven studies (1–13.6%, mainly affecting the greater tuberosity), while humeral subsidences varied from 0% to 97%. Osteolysis rates were investigated in seven studies, with a mean value of 23.5% (range 2.2–59%). Radiological outcomes were stratified based on stem types, revealing variations in humeral stem loosening, lucencies, and osteolysis between the two groups. Among the standard stems, three studies assessed significant humeral stem loosening with a mean value of 3% (range 0–10%). Lucencies around the implants or subsidences were evaluated in four studies, with a mean value of 9.4%. Osteolysis was assessed in two studies, and two studies did not observe this radiological change in their patients, resulting in a mean value of 2.8%. Regarding short stems, all studies evaluated significant humeral stem loosening, with two studies reporting no observed radiological change and a mean value of 5.7% (range 0–33%). Lucencies around the implants or subsidences were assessed in all studies, with a mean value of 25.8% (range 1.9–97%), mainly affecting zones 1/7 or L1. Osteolysis was evaluated in four studies, and one study did not observe this radiological change in their patients, resulting in a mean value of 26%

### 3.3. Clinical Outcomes

The included studies provided various clinical outcomes (Table 3). Within the standard stem group, the ASES score was the most frequently reported, featured in six studies, with an improvement in the mean score from 36.1 preoperative to 74 postoperative. Moreover, in the standard group, the VAS pain score was used in six studies, showing an improvement in the mean value from 6.3 to 1.35. The CS score was assessed in four studies of the standard stem group, with a mean value from 29.5 preoperative and 63.3 post operative. SANE score was evaluated in three studies with an improvement in the mean value from 27.4 to 75.6. Only one study in this group evaluated the overall complications with a value of 13%. In the short stem group, the mean VAS pain score decreased from 6.9 preoperative to 0.85 postoperative (in two studies) and the CS score, evaluated in three studies, registered an improvement from 27.5 preoperative to 71.7 postoperative. In the same group, three studies evaluated the overall complications with a medium value of 4%.

## 4. Discussion

This systematic review provides a detailed comparison of the radiological and clinical outcomes between short and standard humeral stems in cementless RTSA, with a follow-up period of 1 to 5 years. The most important findings of this systematic review are the results of the comparison of the radiological outcomes between uncemented RTSA using short and standard humeral stems. The short stem group exhibited higher rates of radiological outcomes including humeral loosening, lucencies, and osteolysis [24,25]. Contrary to radiographic scores, clinical scores were similar in the different analyzed studies; therefore, they may not significantly impact the clinical outcomes as they do for knee replacements [1,7,13,16].

The findings suggest that both stem types present an acceptable risk of radiological changes post-implantation in patients with osteoarthritis, cuff tear arthropathy, and rheumatoid arthritis.

The integration of RTSA into orthopedic practice has been marked by significant clinical success, addressing a spectrum of conditions from end-stage arthritis to irreparable rotator cuff lesions.

Notably, advancements in implant design and scapular notching reduction have contributed to the reduction in complications over the years [26,27]. The choice between cemented and cementless prosthesis has been an ongoing debate, with uncemented stems gaining popularity due to reduced operative time, the absence of cement-related complications, and ease of revision [8]. The radiological success of cementless RTSA has been evidenced by numerous studies spanning 2 to 10 years [16]. The prevalence of humeral loosening varied across studies, with a slightly higher prevalence noted in those with standard stems. Of the identified radiological changes, subsidence emerged as the most common, reported in 22.86% of all studies (range 0.03–97%). This phenomenon predominantly prevailed in standard stems. The second most frequent radiological change was osteolysis, reported in 23.48% (range 2.2–59%) of all RTSA cases, with a higher prevalence in standard stems. Significant radiographic humeral loosening, primarily of the greater tuberosity, was observed in 6.4% (range 0.4–33%), and the medium values were similar between groups, with 3% in standard stems and 5.65% in short stems [7,28]. In this review, only two studies [21,29] compared the radiological and clinical outcomes of RTSA cementless between standard and short stems. In the study conducted by Merolla et al. [21], the incidence of humeral loosening in the group of patients with the standard stem was 33%, which was higher compared with the short stem group (10%). In the research by Flynn et al. [29], radiographic changes were classified by zone, and the most prevalent observation in the press-fit group was lateral metaphyseal cortical thinning or osteopenia (58%). Mazylerat et al. reported a 43% incidence of patients exhibiting osteolysis during the final radiographic evaluation. Conversely, in the remaining studies, the incidence was notably lower (all below 20%) [19]. These disparities may stem from operator-dependent factors or variations in methods of radiographic data collection. Addressing these differences could serve as a crucial initiative toward standardizing radiographic outcome assessment using universal scoring systems.

All the included studies reported improvements in the clinical measurements (ASES, VAS, CS, SANE). Notably, only four studies (two from short stems group and one from standard stems group) reported complications, and all of them were below 8%. An important concept to consider among the radiological outcomes between short and standard stems in RTSA is the distal filling ratio. It has already been shown that humeral stress shielding in reverse total shoulder prostheses occurs in connection with longer and wider stems and with corresponding further distal force transmission as well as with increased stem-to-humerus filling ratios [18].

As limitations of the study, the included studies were heterogeneous in terms of patient populations. Moreover, the variability in radiographic assessment methods may have influenced the findings. Further research is warranted to address the heterogeneity in patient populations, surgical techniques, and data collection methods. Additionally, this study’s limitations, including potential publication bias and varying study designs, should be acknowledged. Surgeons’ decision-making processes are significantly influenced by a high rate of patient satisfaction.

## 5. Conclusions

Minimal differences in radiological outcomes were found in favor of RTSA implanted with short stems compared with standard stems, primarily represented by subsidences around implants in RTSA with standard stems. However, postoperative clinical outcomes, specifically VAS pain and CS score, were similar between RTSA implanted with standard and short stems. These findings, as a starting point for discussing the clinical correlation concerning radiological changes after cementless reverse shoulder arthroplasty, prove the need for further studies comparing the radiological changes and clinical outcomes to elucidate the long-term implications of this emerging trend in shoulder arthroplasty.

## Figures and Tables

**Table 1:** Demographic characteristics of the included studies.

Study	Year of Publication	Study Type	LOE	No. of Patients	No. of RTSA	Age (Range), y	Follow-Up (m)
Gilot et al. [14]	2015	RCS	III	115	115	75 (70–84)	24–48
Harmsen et al. [13]	2017	CS	IV	219	232	77 (35–89)	24–82
Raiss et al. [1]	2019	CS	IV	72	77	(73–81)	24–58
Broiln et al. [15]	2020	CS	IV	71	71	(73–82)	24–58
Tross et al. [16]	2020	RCS	IV	139	143	(70–85)	12–36
Abduh et al. [17]	2022	RCS	III	66	66	(71–80)	24–50
Erickson et al. [18]	2022	Case–control	III	276	137 + 139	(68–70)	12
King et al. [6]	2014	RCS	III	97	100	71 (55–90)	24–42
Mazaleyrat et al. [19]	2020	RCS	III	70	70	(71–80)	9 y (5–20 y)
Denard et al. [20]	2019	RCS	III	93	93	(48–88)	24
Merolla et al. [21]	2017	RCS	III	74	74	(73–81)	24
Kim et al. [22]	2021	RCS	III	104	104	(74–83)	24–60
Wiater et al. [23]	2014	RCS	III	64	64	72 (48–92)	24–63

CS: case series; RCS: retrospective cohort study; LOE: level of evidence; RTSA: reverse total shoulder arthroplasty; y: years; m: months.

**Table 2:** Radiological outcomes of the included studies.

Study	Diagnosis	Type of Implant	Radiographic Humeral Stem Loosening	Lucencies Around Implants or Subsidence	Osteolysis
Gilot et al. [14]	Rotator CTA	RTSA Equinoxe prosthesis (Exactech Inc.) standard stem press-fit	0%	30	N.R.
Harmsen et al. [13]	CTA, Massive rotator cuff tear, Primary OA with posterior glenoid bone loss, RA, Primary OA with rotator cuff tear	RTSA diaphyseal standard stem press-fit	0.4%	Z2 and Z6: 6%; Z1 and Z7: 97%	N.R.
Raiss et al. [1]	OA, CTA	Ascend flex RTSA short stem	N.R.	15%	N.R.
Broiln et al. [15]	OA	RTSA uncemented Standard stem (De Puy/Tornier Aequalis/Zimmer Trabecular Metal)	1.4%	Z1 and Z7: 41–44%, Others: 19%	Z1: 15%; Z7: 16%; Others: 2–6%
Tross et al. [16]	Primary OA, CTA	Short stem RSA (Ascend^TM^ Flex)	N.R.	11%	N.R.
Abduh et al. [17]	Primary OA, CTA	Ascend Flex stem (Tornier SAS) short stem	N.R.	N.R.	9%
Erickson et al. [18]	Primary OA	Apex (short stem) RTSA	2.2%	1.5%	2.2
		Univers (standard stem) RTSA	5.6%	2.2%	12.9%
King et al. [6]	Primary RTSA	Uncemented Exactech Equinoxe standard stem	2%	3.9%	N.R.
Mazaleyrat et al. [19]	Primary RTSA	Press-fit standard stem (Aequalis Reversed Tornier or Delta III DePuy)	1%	Z1: 4%; Z2: 1%; Z7: 3%	59%
Denard et al. [20]	Rotator cuff arthropathy, primary OA, failed cuff repaired	Univers Revers; Arthrex, Standard stem	1.5%	Z1: 53.8%; Others: 6.45%	43%
Merolla et al. [21]	CTA	Aequalis Reversed II standard stem	33%	25%	N.R.
		Aequalis Ascend Flex short stem	10%	10%	N.R.
Kim et al. [22]	OA, CTA	Equinoxe, Exactech press-fit metaphyseal, grit-blasted humeral standard stem	13.6%	1.9%	N.R.
Wiater et al. [23]	CTA, massive rotator cuff tear	Zimmer standard stem (Trabecular Metal Reverse Shoulder)	0%	3%	0%

CTA: cuff tear arthropathy; RTSA: reverse total shoulder arthroplasty; N.R.: not reported; OA: osteoarthritis; RA: rheumatoid arthritis.

**Table 3:** Clinical outcomes of the included studies.

Study		VAS		ASES		CS		SANE		Overall Complication Rate
		Pre	Post	Pre	Post	Pre	Post	Pre	Post	
Harmsen et al. [13]		6.5	0.9	32.2	77.5			23.4	76.7	
Raiss et al. [1]										7.8
Tross et al. [16]							72.2			4.2
Abduh et al. [17]			Improvement				Improvement			0
Erickson et al. [18]	Apex	5.4	0.9	40.2	85.6			30.2	77.3	
	Univers	6	1.5	37.9	78.5			32.3	71.4	
King et al. [6]				32.7	75.3	33.9	69.4			13
Denard et al. [20]			1.5	37.9	74.7			26.4	78.8	
Merolla et al. [21]	Aequalis	8.4	0.9			17.9	69.6			
	Ascend Flex	8.5	0.8			27	71.2			
Kim et al. [22]			2	40	61	36.8	50			
Wiater et al. [23]		6	1.3	36.1	77.1	29.2	64.1			

## Data Availability

No new data were created or analyzed in this study. Data sharing is not applicable to this article.

## Abbreviations

The following abbreviations are used in this manuscript:

ASES	American Shoulder and Elbow Surgeons
CS	Constant Score
CTA	Cuff tear arthropathy
OA	Osteoarthritis
PRISMA	Preferred Reporting Items for Systematic Reviews and Meta-Analyses
RA	Rheumatoid arthritis
RCT	Randomized controlled trials
RTSA	Reverse total shoulder arthroplasty
SANE	Single Assessment Numeric Evaluation
VAS	Visual Analog Scale
